# Assessment of Physicochemical Properties of Orange Juice Concentrate Formulated with Pectin, Xanthan, and CMC Hydrocolloids

**DOI:** 10.1155/2024/7013553

**Published:** 2024-05-03

**Authors:** Mehrdad Mohammadi, Mina Mahdavi-Yekta, S. Fatemeh S. Reihani, Nasim Khorshidian, Mahdi Habibi, Amin Mousavi Khaneghah

**Affiliations:** ^1^Department of Food Technology Research, National Nutrition, and Food Technology Research Institute, Faculty of Nutrition Sciences and Food Technology, Shahid Beheshti University of Medical Sciences, Tehran, Iran; ^2^Department of Animal and Veterinary and Food Science, University of Idaho, Moscow, ID, USA; ^3^Faculty of Biotechnologies (BioTech), ITMO University, 9 Lomonosova Street, Saint Petersburg, 191002, Russia

## Abstract

Orange concentrate (OC) is one of the main raw materials in the nonalcoholic beverage industry. Considering the difference in orange varieties, preserving its natural quality is essential to yield a product with favorable attributes and physical stability. Thus, the present study is aimed at assessing the effect of pectin, xanthan, and carboxymethyl cellulose (CMC) in a concentration range of 0–0.2% (*w*/*v*) along with mixing temperature on Brix, pH, acidity, density, turbidity, and viscosity of OC and at calculating the model equation for each attribute. The results showed that, except for CMC, the influence of concentration, type, and amount of hydrocolloid on pH changes was insignificant. Adding each hydrocolloid individually, in pairs, or threes reduced the density, and the measured density was lower at a mixing temperature of 4°C. Also, it was observed that mixing temperature was the only factor influencing turbidity, and the values were significantly lower at 80°C compared to 4°C. A significant interaction effect of xanthan concentration and mixing temperature on the Brix was observed. Adding hydrocolloids, except pectin, resulted in a significant (*p* < 0.05) increase in viscosity, and xanthan had the greatest effect on the viscosity. A suitable model was designed using pectin and xanthan, pectin and CMC, and all three gums, resulting in a final OC product with high stability and improved physical and chemical attributes. The optimized values for Brix, pH, acidity, density, turbidity, and OC viscosity were achieved using 0.08% pectin, 0.19% xanthan, and 0.08% CMC at 80°C mixing temperature.

## 1. Introduction

Due to the growth in consumers' health awareness, fruit beverages are now the second largest nonalcoholic segment due to their functional and health-promoting properties [1]. Daily consumption of fruits and juices is associated with a reduced risk of cancer and cardiovascular diseases due to the presence of antioxidant compounds, vitamins, and minerals [2–4].

Orange is an excellent source of vitamin C, providing 64% of an individual's daily requirement [5]. In addition, it is rich in folic acid and potassium, an excellent source of bioactive compounds, and is an important commodity in most countries [6]. Among fruit beverages, orange juice concentrate (OC) is one of the main raw materials used in this industry [7, 8].

In the producing countries, oranges are mostly consumed fresh, and their production is not adapted to the beverage industry. This leads to the import of OC from other countries. Therefore, OC stability would be key to maintaining imported beverages' natural quality and taste. Considering that orange varieties can affect the quality characteristics of the resulting concentrate, food hydrocolloids must be used to ensure the stability of the final products [9, 10]. Hydrocolloids are widely used in most beverages due to their favorable contribution to maintaining and/or improving the original texture, physical and chemical characteristics, and rheological properties [11–13].

As a linear polymer, pectin is one of the main constituents of plant cell walls. The major component is a polymer of galacturonic acid, some of whose carboxyl groups are esterified with methanol. The emulsifying properties of pectin are probably due to the protein residues associated with arabinogalactan and the hydrophobic characteristics of acetyl and methyl esters despite its overall hydrophilic attribute [14–17]. The molecular weight of pectin, degree of acetylation, and degree of esterification are the main factors that determine pectin's mechanism of action and gel properties [18].

An inexpensive derivative of cellulose, namely, carboxymethyl cellulose (CMC), as another water-soluble hydrocolloid is widely used in the food and beverage industry as a thickener and transparent coating with strong adhesion and mechanical properties [19–21]. In a recent study on the influence of different hydrocolloids on cloudy strawberry beverages, samples with CMC at a dose of 0.2% showed the highest score in overall taste evaluation and a favorable consistency compared to guar gum and locust bean gum. Another research study indicated that 1% CMC added to tamarind juice resulted in an acceptable consistency at room temperature [22].

Xanthan gum is a high molecular weight extracellular heteropolysaccharide produced by *Xanthomonas* through submerged aerobic fermentation in a sterile medium. Xanthan comprises *β* (1-4) linked D-glucose, and its main constituents include glucose, mannose, and galacturonic acid units. It is extensively used in the food industry due to its remarkable thickening, emulsifying, stabilizing, and foaming characteristics, as well as its high stability over a wide range of pH and temperature [23–25].

Depending on the target product, these hydrocolloids can be used alone or in combination [26]. For instance, a combination of different stabilizers in the formulation of orange juice and acidified protein drinks was used by Mirhosseini et al. [16] and Liu et al. [27], respectively, to enhance their storage stability in terms of physical and sensory characteristics [16, 27]. In addition, Staubmann et al. [28] assessed the influence of three different hydrocolloids, i.e., pectin, locust bean gum, and guar gum, on the long-term storage stability of cloudy orange juice ready-to-drink beverages. They confirmed the greater effect of combined use compared to single use [28].

The growing knowledge of phase behavior and interactions of hydrocolloid mixtures at the molecular level has stimulated interest in discovering novel synergistic combinations of hydrocolloids [29].

The products based on orange concentrate are classified and defined according to different standards. These products include syrups, nectars, juices, and drinks, in which the amount of concentrate and Brix differ. Therefore, assessing the physical and chemical properties of the concentrate added with the selected hydrocolloids and creating balanced formulations can help to achieve stable quality in all these different OC-based products.

Therefore, this study is aimed at evaluating the synergistic effects of adding three hydrocolloids, including pectin, xanthan, and CMC, on physicochemical properties of OC such as Brix, pH, acidity, density, turbidity, and viscosity. Moreover, the optimum condition for achieving the synergistic effect was calculated, and a regression model was designed to obtain a final product with favorable stability, minimum turbidity, and maximum density and viscosity.

## 2. Materials and Methods

### 2.1. Chemicals and Reagents

All chemicals and reagents were of analytical grade and purchased from Merck (Darmstadt, Germany). High methoxyl pectin, xanthan, and CMC were purchased from Danisco (Denmark). OC was purchased from Khazarnoosh (Iran), and sugar was obtained from Amirkabir Co. (Iran).

### 2.2. Preparation of Hydrocolloid Solution

Danisco's product specifications instructed that pectin, xanthan, and CMC gums can be used at both 4°C and 80°C. To prepare the hydrocolloid solutions, they were mixed with sugar in a 1 to 3 ratio and then dissolved in a given amount of water at the specified temperature using a laboratory mixer to obtain a homogeneous solution. Subsequently, they were refrigerated for 24 hours to obtain a bubble-free solution. According to the test design, the maximum amount of the three gums added to the concentrate is 0.6% [30]. Therefore, a standard solution was prepared for each gum to minimize the changes in Brix. Accordingly, 48 centrifuge tubes were used for 24 series of experiments.

### 2.3. Preparation of Hydrocolloid Mix and OC

According to the experimental design (see Table 1), hydrocolloids in a 0–0.2% (*w*/*v*) concentration were added individually, in pairs, or threes into 48 centrifuge tubes. Therefore, from standard solutions, 1 mL of 5% (*w*/*v*) pectin solution, 1.5 mL of 5% (*w*/*v*) xanthan solution, and 1 mL of 5% (*w*/*v*) CMC solution were added to 50 g of OC [31].

### 2.4. Physical and Chemical Properties of OC

Brix and pH were measured at 25°C using a refractometer (KRUSS, Germany) and a digital pH meter (WTW, Germany). Acidity was measured by using a digital burette (VWR, UK) based on titration by 0.1 N NaOH according to values defined by rule no. 2685, Institute of Standards and Industrial Research of Iran (ISIRI) [32], and expressed as grams of citric acid per 100 mL of sample. Density was measured at 25°C using a 25 mL pycnometer (Merck, Germany). To measure turbidity, distilled water was added to each sample until it reached 12° Brix, and turbidity was determined using a digital turbidity meter (LUTRON, Taiwan). Viscosity was also measured using a Brookfield viscometer (Fisher, USA). The viscosity of samples was read 20 s after spindle rotation at two speeds of 20 and 25 rpm at 25°C [33].

### 2.5. Statistical Analysis

The response surface methodology was used to analyze the data and select the values between the minimum and maximum concentrations of gums. This model also considered the interaction and quadratic power of the concentrations. The generalized polynomial model proposed is shown in

(1) $$\begin{array}{c} F=\alpha \times A+\beta \times B+\chi \times C+\delta \times D+\varepsilon \times AB+\varphi \times AC+\phi \times AD+\gamma \times BC+\eta \times BD+\iota \times CD+\kappa \times A^{2}+\lambda B^{2}+\mu C^{2}. \end{array}$$

*A* represents the pectin concentration, *B* represents the xanthan concentration, *C* represents the CMC concentration, and *D* represents the mixing temperature. After the experimental tests, fitting calculations were performed on the results.

The significance of the equation parameters for each response was analyzed by analysis of variance (ANOVA) and also by *F*-ratio at a probability (*P*) of 0.05 [34]. The models' adequacy was assessed using model analysis, lack-of-fit test, and determination coefficient (*R*^2^) analysis. Moreover, the response surface method was able to estimate the optimized conditions for the preparation of OC samples with high desirability (0.95).

## 3. Results and Discussion

### 3.1. Acidity and pH

Table 1 presents the results of the physical and chemical properties of fresh OCs (pH, acidity, turbidity, Brix, and viscosity) before and after the addition of pectin, xanthan, and CMC in random values obtained from a statistical design (RSM).

The results showed that except for CMC, the influence of concentration, type, and amount of hydrocolloid on pH changes was insignificant. The model obtained for pH variation by changing the type, amount, and mixing temperature of each hydrocolloid added to OC is as shown in

(2) $$\begin{array}{c} \text{pH}=2.96-0.019A-0.01B+0.033C-\left(8.86e-003\right)D-0.013AB-0.022AC-0.023AD+0.036BC+0.018BD-\left(6.604e-004\right)CD+0.053\left(A^{2}\right)+\left(5.072e-03\right)B^{2}-0.031\left(C^{2}\right). \end{array}$$

In a study by Nwaokoro and Akanbi [35], the addition of hydrocolloids such as xanthan gum and carboxyl methylcellulose (CMC) into tomato-carrot juice had no significant effect on the titratable acidity of the juice. Similar results have been reported by Anggrahini and Pratama [36], in which commercial CMC and snake fruit kernel CMC at levels of 0.1, 0.3, and 0.5% to snake fruit syrup did not affect pH. Nevertheless, Ghannadi et al. reported a different result when conducting a similar study, in which the adding pectin (0.2 and 0.3%) and xanthan gum (0.1 and 0.2%) to orange juice with pulp showed a significant influence on pH by adding 0.2% xanthan [37]. Similarly, adding 0.5% CMC to red guava syrup has been reported to increase pH values [38]. This agrees with the results obtained by Izadi and Djomeh [39], who reported an increase in pH value in a model beverage emulsion by increasing CMC concentrations due to a high number of carboxyl groups. Also, Mousa [40] reported increased pH in cloudy guava juice containing Arabic gum and CMC due to sodium ions in CMC.

### 3.2. Density

According to the analysis of variance, the effect of pectin concentration and mixing temperature on density changes was significant (*p* < 0.05). An *F*-ratio of 2.87 indicated that the model had a probability of error of 5.1%, so the possibility of instrumental error was very high.

A lack-of-fit value of 0.62 and *R*^2^ of 0.8 indicated a relatively good model fit. The model obtained for density variation by changing the type, amount, and mixing temperature of each hydrocolloid added to OC is given as

(3) $$\begin{array}{c} \begin{array}{c} \text{Den}=1.15-0.04A-.019B+0.019C-0.031D-0.022AB+\left(3.701e-003\right)AC-0.011AD+0.015\\ BC-\left(6.775e-003\right)BD+0.017CD+0.034\left(A^{2}\right)+0.036\left(B^{2}\right)-0.027\left(C^{2}\right). \end{array} \end{array}$$

**Figure 1:**
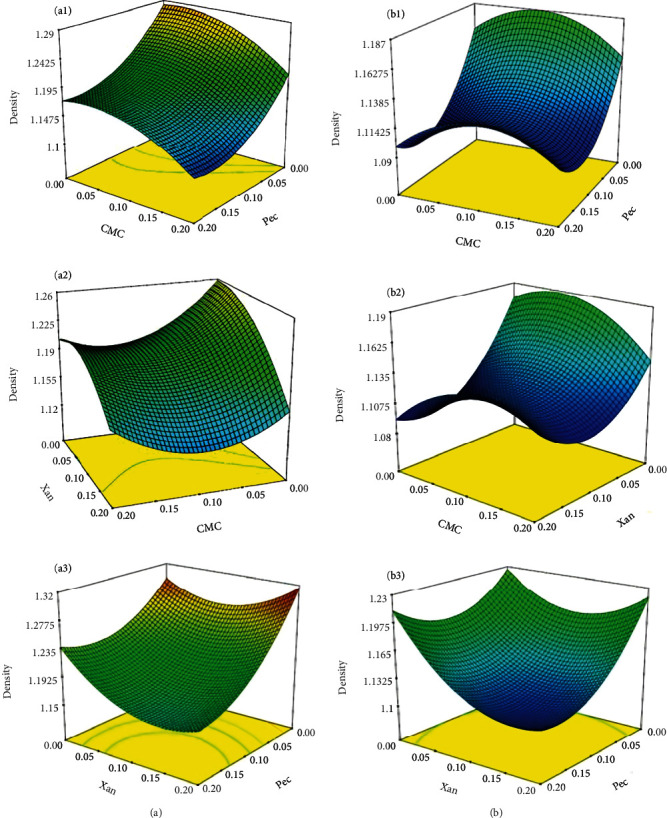
Surface plots of interactive effects of independent variables on density of OC at (a) 4°C and (b) 80°C.

As shown in Figure 1, adding each hydrocolloid individually, in pairs, or threes reduced the density. All results showed decreased density when hydrocolloids were added in the defined concentration range. Considering that the amount of dry matter added to the formulation was much less than the amount of water consumed and that a 20° Brix solution was added to a 62.5° Brix solution, the decrease in density is well justified.

In other words, by adding 0.2% pectin and 0.2% xanthan, an OC with a density of 1.255 g/cm^3^ was obtained. Furthermore, the mixing temperature had a significant effect on the change in density (*p* < 0.05), probably because dispersion and dissolution of hydrocolloids are directly related to temperature [24, 41, 42]. Thus, a more homogeneous solution is obtained at higher temperatures, and the measured density was lower at a mixing temperature of 4°C. Similarly, the descriptive model of pectin became significant (*p* < 0.05) due to the higher degree of dissolution of pectin in the OC.

The plots obtained from the descriptive model of density changes indicated that proper dissolution and particle dispersion play an important role in interpreting the results [29]. For example, as shown in Figure 1, density decreases with increasing xanthan and CMC concentrations while pectin concentrations remain constant. Overall, the final results confirmed that hydrocolloids decrease OC density. This decrease cannot prevent monophasic liquid systems such as syrups from becoming biphasic; in these systems, white sedimentation occurs due to the difference in density of the sugar syrup and OC as the two dominant phases. In other words, due to the similarity in density of these two phases, the OC flow rate of OC towards the surface would be slower.

### 3.3. Turbidity

According to the results, mixing temperature is the only factor influencing turbidity (*p* < 0.05). A lack of fit of 4.23% and an *R*^2^ of 0.74 indicated a significant lack-of-fit model.

The model obtained for the turbidity by varying the type, amount, and mixing temperature of each hydrocolloid added to OC is presented in

(4) $$\begin{array}{c} \text{Ntu}=452.58-14.34A-7.8B+5.92C-50.14D-4.19AB+17.94AC+13.42AD+8.35BC+17.5BD+21.47CD+44.52\left(A^{2}\right)+20.92\left(B^{2}\right)-48.5\left(C^{2}\right). \end{array}$$

**Figure 2:**
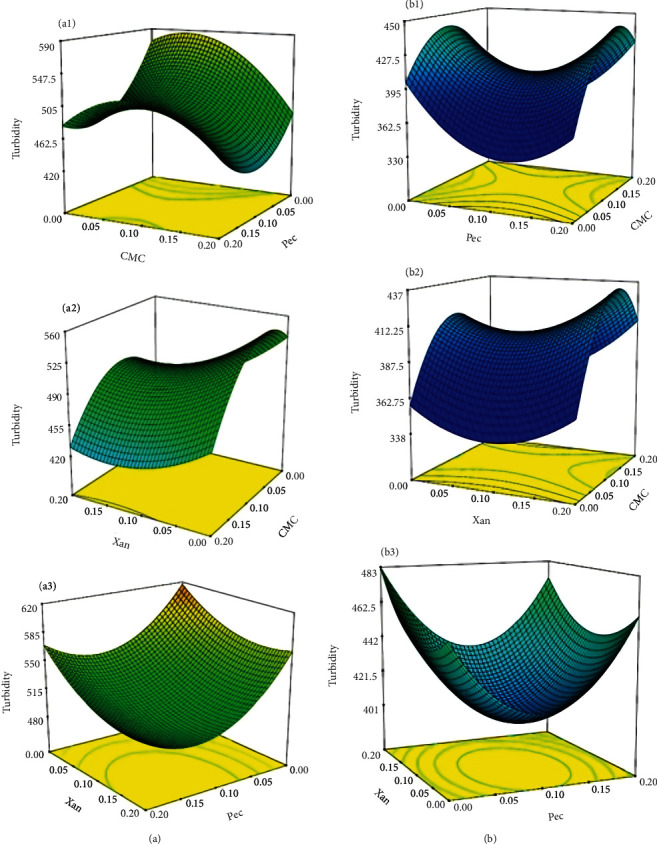
Surface plots of interactive effects of independent variables on turbidity of OC at (a) 4°C and (b) 80°C.

In all plots in Figure 2, turbidity values at 80°C of mixing temperature were significantly lower than those obtained at four °C (*p* < 0.05). Hydrocolloids can cover oil droplets, inhibit droplet flocculation, and, ultimately, affect the mean particle size in the dispersed phase [43]. Generally, the turbidity of an emulsion system depends on parameters such as dispersed phase density, transmitted light, mean particle size, and distribution [44, 45]. In this study, the obtained model confirmed that a higher mixing temperature significantly reduced the turbidity (*p* < 0.05). This can be justified by the fact that in a system with the presence of hydrocolloid, the Brownian motion of large molecules slows down as water is trapped within the molecular structure of the hydrocolloid, which negatively affects the turbidity caused by the motion of large molecules [46].

While all hydrocolloids used are water-soluble and transparent, pectin showed the highest reducing effect on turbidity due to its molecular weight. The molecular weight of the hydrocolloid directly affects the adsorption density, which in turn affects turbidity and viscosity [47–49]. A combination of electrostatic and steric interaction forces are the two mechanisms involved in hydrocolloid stabilization [29]. It has also been stated that electrostatic repulsion between negatively charged hydrocolloids and the juice particles may lead to a stable cloud by influencing the turbidity and sedimentation behavior, as reported for other juice beverages [28]. In a study by Ozgur et al. [42], it was observed that an increase in CMC level in the hydrocolloid mixture added to the citric acid-sucrose model system provided a lower increment in the turbidity values compared to xanthan and pectin because CMC was tasteless, odorless, and formed clear solutions without cloudiness or opacity.

At a concentration of 0.1%, pectin, xanthan, and CMC exhibited turbidity values of 357, 401, and 454 NTU, respectively. Physical properties of orange beverages with pectin and CMC added were studied during 6 months of storage by Mirhosseini et al. [16], and samples with 4.5% and 1.5% (*w*/*w*) pectin showed the highest and lowest stability, respectively. According to their results, the turbidity of all orange beverage samples decreased after two months of storage, indicating a loss of stability for six months [16].

According to a report by Genovese and Lozano [50], the turbidity of noncentrifuged cloudy apple juice was stabilized by adding 0.4-0.5% (*w*/*w*) xanthan or CMC for prolonged storage. They also demonstrated that CMC stabilized apple juice cloudiness better than xanthan, perhaps due to the higher electronegativity of CMC, which increases particle-particle repulsion. Similarly, in another report, the incorporation of 0.3% CMC and 0.2% sodium alginate had a significant effect on the cloud stability of the litchi juice [45].

### 3.4. Brix and Viscosity

**Figure 3:**
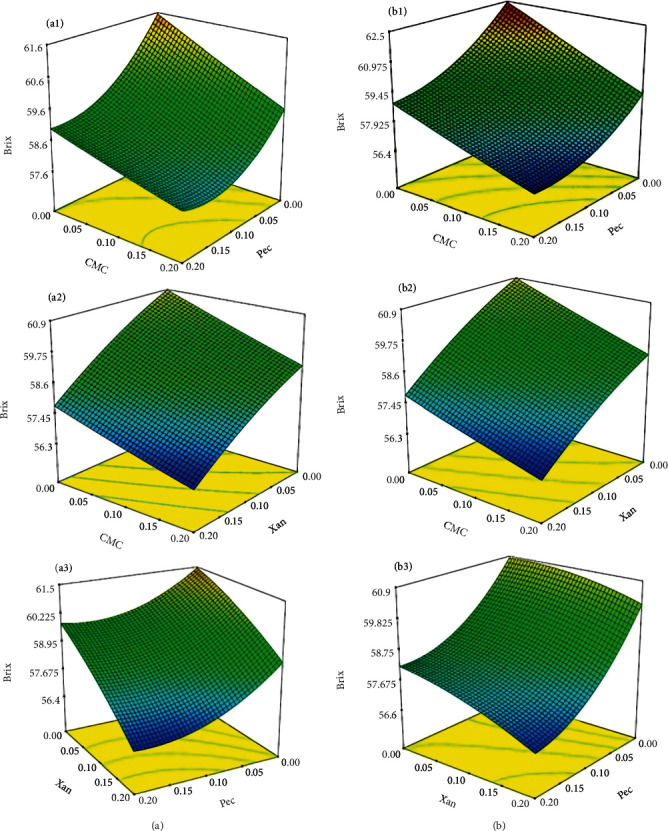
Surface plots of interactive effects of independent variables on Brix of OC at (a) 4°C and (b) 80°C.

The effect of hydrocolloid type and concentration on Brix was significant (*p* < 0.05) only when used separately (see Figure 3). In addition, a significant interaction effect of xanthan concentration and mixing temperature on the Brix was observed (*p* < 0.05). However, the other hydrocolloids' different mixing temperatures and synergistic effects were insignificant. In addition, an *F*-ratio of 15.37 and a probability of error of 0.01% indicated the significance of the model concerning Brix. A lack of fit of 3.72 indicates a probability error of 8.77%, while *R*^2^ = 0.95 indicates an acceptable goodness of fit. The general model obtained for the variation in Brix by varying the type, amount, and mixing temperature of each hydrocolloid mixed with OC is given by

(5) $$\begin{array}{c} \text{Bx}=58.86-1.26A-1.0B-1.15C-0.11D-0.19AB+26AC+0.25AD+0.11BC+0.45BD-0.32CD+0.73\left(A^{2}\right)-0.23\left(B^{2}\right)+0.032\left(C^{2}\right). \end{array}$$

According to the plots in Figure 3, at constant pectin and xanthan concentrations, a linear decrease in Brix can be attributed to changes in CMC concentration [16]. Based on the analysis of variance, changes in the xanthan and CMC concentration and mixing temperature significantly influenced the viscosity of OC (*p* < 0.05). The *F*‐ratio value = 8.66 indicated the significance of the model with only a 0.08% chance of error (*p* < 0.01). In addition, a lack of fit of 3.42 and a probability of error of 10.16% indicated an acceptable goodness of fit.

The model obtained for viscosity variation by varying the type, amount, and mixing temperature of each hydrocolloid added to OC is as given in

(6) $$\begin{array}{c} \text{Vis}=7.12-\left(1.86e-003\right)A+1.48B+1.31C+0.91D+0.27AB-0.0066AC+0.011AD-0.25BC+0.41BD-0.057CD+0.25\left(A^{2}\right)-0.59\left(B^{2}\right)-0.18\left(C^{2}\right). \end{array}$$

**Figure 4:**
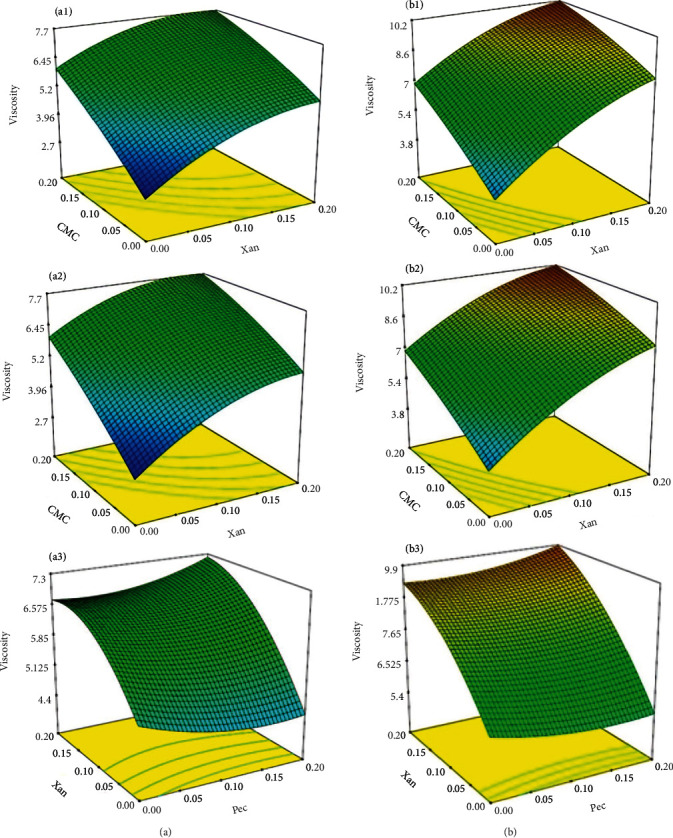
Surface plots of interactive effects of independent variables on viscosity of OC at (a) 4°C and (b) 80°C.

As shown in Figure 4, at a constant pectin concentration (0.1%), the highest viscosity was observed in OC samples with added xanthan and CMC at 80°C. However, adding pectin (0–0.2% *w*/*v*) had no significant effect on the viscosity. Considering the slope of the curves related to the effect of xanthan concentration (0–0.2%), it is confirmed that this hydrocolloid had the highest effect on viscosity. Xanthan gum is a branched hydrocolloid with more branches and longer branches than those in the other types of gums, which means that it can form many hydrogen bonds and greatly increase the viscosity [51]. It can interact synergistically with other polysaccharides (e.g., cellulose and its derivatives, guar gum, and locust bean gum) and synthetic polymers to enhance the viscosity of aqueous solutions. Xanthan can form a complex network by conforming to a multiple helix (single, double, and triple) with strong intermolecular linkages [52].

The results showed that the combination of pectin, xanthan, and CMC significantly affected viscosity. However, their synergistic effect still needs to be confirmed.

Adding hydrocolloids, except pectin, resulted in a significant (*p* < 0.05) increase in viscosity. Similarly, in a recent study by Aggarwal et al. [53], the highest viscosity of kinnow juice was obtained with CMC and pectin, respectively [53]. Liu et al. [27] also used pectin and CMC in combination with guar, locust bean, and gellan gums to evaluate the combined effect of stabilizers on acidified protein drinks [27]. According to their report, pectin/guar, pectin/locust bean gum, and CMC at 0.33/0.22% *w*/*w* resulted in the highest viscosity in the samples without any change in flavor. Another study on mulberry juice indicated that adding xanthan gum to mulberry juice yielded higher viscosity and better physical properties than CMC [51]. Similarly, in our study, CMC and xanthan had a better effect on OC viscosity than pectin. According to Izadi and Djomeh [39], polymeric chains of CMC at high concentrations or molecular weight became entangled in the model emulsions, and the bulk flow was difficult. Therefore, the viscosity significantly increased.

In fresh OC, Brix and viscosity were measured as 62.7° and 2.37 Pa·s, whereas with the addition of 0.2% pectin, xanthan, and CMC, the values of Brix and viscosity were as 56.5° and 10.22 Pa·s, respectively. As mentioned above, the molecular weight and the number of suspended particles influence the viscosity at constant concentrations. Therefore, due to the high molecular weight and large particle size of hydrocolloids, an increase in viscosity can be observed as the Brix decreases [54–56]. When these three hydrocolloids were used individually at the same concentration (0.2%), the viscosity values were 2.64, 6.85, and 7.69, adding pectin, CMC, and xanthan, respectively. As expected, xanthan had the greatest effect on the viscosity. This may be due to the high molecular weight, reduced shear rate, and viscoelastic properties of xanthan gum. Similar results were reported that xanthan had a more increasing effect on the viscosity of mango juice and mango starch kernel compared to CMC and guar gum, respectively [16, 57, 58].

The synergistic effect of pectin was not significant when combined with other hydrocolloids, i.e., xanthan and CMC. For instance, 0.1% pectin and xanthan resulted in a viscosity of 6.6 Pa·s lower than that of xanthan alone. Meanwhile, with a higher concentration (0.2%) of pectin and xanthan, the viscosity was slightly higher (8.7 Pa·s) than that of xanthan alone (8 Pa·s). This result is in agreement with a report by Mousa [40], in which the effect of the binary use of three hydrocolloids, i.e., CMC, gum Arabic, and pectin, was studied and the best combination with the best result did not include pectin. It was found that juice containing CMC had higher viscosity compared to samples containing pectin due to the higher molecular weight of CMC [40].

As indicated by the model fit, the pectin concentration range was considered unsuitable since no significant effect occurred. A 0.3-0.5% pectin concentration would be more influential, although it could be more practical due to its high cost [14]. Moreover, the combination of 0.1% xanthan and CMC resulted in a favorable synergistic effect on viscosity (8.26 Pa·s), which may be because they carry the same negative charges that lead to coagulation and, ultimately, an increase in viscosity. The free movement of the hydroxyl groups in the solution increases their affinity for water molecules to form a hydrophilic compound, leading to swelling and increased viscosity [59]. However, this effect was not considered significant in the resulting model. In fact, due to the low pectin content of oranges provided at the end of the season, using pectin in fruit concentrate is necessary to prevent the action of pectin methyl esterase.

Combining these three hydrocolloids could improve the viscosity at a more reasonable cost. In addition, the hot mixing method was preferred due to the positive effect of temperature on the final viscosity (*p* < 0.05) and more effective dissolution and dispersion.

### 3.5. Optimal Values

Using the models obtained based on experimental results, optimal values were calculated at a confidence level of 95%, in which the addition of 0.08% pectin, 0.19% xanthan, and 0.08% CMC (*w*/*v*) to the OC at 80°C resulted in a final product with the following attributes: Brix = °60, density = 1.27 g/cm^3^, pH = 2.94, acidity = 11.9%, turbidity = 425 NTU, and viscosity = 9 Pa·s, among which viscosity and turbidity were higher and lower, respectively, compared to the initial values defined by ISIRI rule no. 2685 [32].

## 4. Conclusions

The present study demonstrated that adding hydrocolloids influenced different physicochemical properties of orange juice concentrate. Among the hydrocolloids, only CMC had a significant effect on pH changes. Regarding density, it was demonstrated that the addition of hydrocolloids and a mixing temperature of 4°C had a reducing effect on density values. The OC samples prepared at 4°C showed higher turbidity than 80°C, and the effect of adding hydrocolloids was insignificant. All hydrocolloids had a significant effect on Brix changes in OC when used individually, but the interaction effect of mixing temperature and hydrocolloids was insignificant except for xanthan. Incorporating xanthan and CMC increased viscosity, and xanthan was more effective in this regard. Based on the experimental results, a model was defined using pectin and xanthan, pectin and CMC, and all three gums, resulting in a final OC product with high stability and improved physical and chemical properties. As a result, the hydrocolloid concentration can be determined from their physical and chemical properties using this defined model, and vice versa. The results of this research study can be implemented in beverage formulation, especially orange nectar and syrup. Further studies are recommended to investigate the effects of different hydrocolloids to optimize the formulation of other types of beverages and products.

## Figures and Tables

**Table 1:** RSM design matrix with experimental values of independent (pectin, xanthan, CMC, and temperature) and response (pH, acidity, turbidity, Brix, and viscosity) variables.

Run	*A*—pectin (% *w*/*v*)	*B*—xanthan (% *w*/*v*)	*C*—CMC (% *w*/*v*)	*D*—temperature (°C)	pH	Acidity (%)	Density (g/cm^3^)	Turbidity (NTU)	Brix (°)	Viscosity (Pa·s)
Control^a^	0	0	0	0	3	12.79	1.325	666	62.7	2.31
1	0	0.2	0	80	2.93	11.90	1.225	469	62	8.11
2	0	0.2	0.2	4	3.08	11.05	1.255	478	57.8	7.41
3	0	0.08	0.2	80	3.01	11.59	1.185	421	59.5	9.08
4	0	0	0.2	4	3.05	11.40	1.235	505	60.8	6.85
5	0	0	0	4	3.00	12.79	1.325	666	62.7	2.31
6	0	0.13	0	4	2.90	11.86	1.272	508	61	6.38
7	0	0.2	0	80	2.95	11.72	1.141	409	61.5	7.69
8	0	0.08	0.2	80	3.04	11.55	1.112	395	58.2	9.07
9	0.05	0.09	0.08	80	3.10	11.90	1.147	358	60	7.99
10	0.08	0	0	80	2.90	12.57	1.192	354	61	3.80
11	0.1	0.15	0.1	4	2.95	11.80	1.245	610	58.8	5.26
12	0.1	0.01	0.08	4	2.93	11.85	1.185	519	58.7	5.26
13	0.12	0.2	0.2	80	3.00	11.70	1.131	391	56.2	10.98
14	0.12	0.2	0.2	4	2.94	11.65	1.115	416	59.3	8.93
15	0.13	0.07	0.2	4	2.96	11.45	1.098	410	58	6.96
16	0.2	0	0	4	3.10	12.40	1.285	540	61.1	2.64
17	0.2	0.2	0.2	4	3.03	11.09	1.131	453	56	9.14
18	0.2	0.11	0	80	2.91	12.20	1.084	400	58.4	7.19
19	0.2	0	0.09	80	2.94	12	1.192	439	58.1	6.11
20	0.2	0.2	0	4	2.89	13.57	1.212	483	56.9	5.91
21	0.2	0	0.2	80	2.95	11.75	1.186	468	57	6.23
22	0.2	0.13	0.13	80	2.94	11.81	1.132	436	57.4	8.68
23	0.2	0.2	0.2	80	2.93	11.03	1.118	470	56.4	10.61
24	0.2	0.2	0	4	2.94	11.31	1.096	416	57	6.07

^a^Orange concentrate without pectin, xanthan, and CMC is considered as control.

## Data Availability

The data used to support the findings of this study are included within the article.
